# Using Anchoring Vignettes to Adjust Self-Reported Personality: A Comparison Between Countries

**DOI:** 10.3389/fpsyg.2018.00325

**Published:** 2018-03-14

**Authors:** Selina Weiss, Richard D. Roberts

**Affiliations:** ^1^Department of Individual Differences and Psychological Assessment, Institute of Psychology and Education, Ulm University, Ulm, Germany; ^2^ProExam - an ACT Affiliated Company, New York, NY, United States

**Keywords:** anchoring vignettes, personality scales and inventories, Big Five, differential item functioning, cross-cultural differences

## Abstract

Data from self-report tools cannot be readily compared between cultures due to culturally specific ways of using a response scale. As such, anchoring vignettes have been proposed as a suitable methodology for correcting against this difference. We developed anchoring vignettes for the Big Five Inventory-44 (BFI-44) to supplement its Likert-type response options. Based on two samples (Rwanda: *n* = 423; Philippines: *n* = 143), we evaluated the psychometric properties of the measure both before and after applying the anchoring vignette adjustment. Results show that adjusted scores had better measurement properties, including improved reliability and a more orthogonal correlational structure, relative to scores based on the original Likert scale. Correlations of the Big Five Personality Factors with life satisfaction were essentially unchanged after the vignette-adjustment while correlations with counterproductive were noticeably lower. Overall, these changed findings suggest that the use of anchoring vignette methodology improves the cross-cultural comparability of self-reported personality, a finding of potential interest to the field of global workforce research and development as well as educational policymakers.

## Introduction

Self-report questionnaires are a dominant assessment methodology in the social sciences. They are used to estimate important information about a participant's personality, attitudes, values, and beliefs. However, self-report questionnaires are prone to various biases that challenge the utility of this methodology including the validity of the data. These biases include cultural artifacts (e.g., measurement artifacts and differences in response sets due to differences in communication styles between cultures; Van de Vijver and Leung, 1997; Fischer, 2004), active deception (e.g., Ziegler et al., 2011), and personal biases in response styles such as extreme responding, midpoint responding, and acquiescent (i.e., a tendency to agree with items and hence only using the upper half of the response option scale) and disacquiescent responding (i.e., a tendency to generally disagree with items and hence only use the bottom half of the response option scale; Van Vaerenbergh and Thomas, 2013). Cross-cultural biases occur because participants compare themselves to the standards and values of their cultural group, also known as their reference group (Peng et al., 1997; Heine et al., 2002). The difference in item responses between the two groups is called *Differential Item Functioning* (DIF; Holland and Wainer, 1993; Osterlind and Everson, 2009). There are several reasons why an item, used in a cross-cultural context, can show DIF (Ellis et al., 1993). An item can display DIF because of (1) mistakes in the item's translation, (2) because participants ascribe unique meanings to the item because of their culture, or (3) participants have different cultural knowledge (Johnson et al., 2008). The anchoring vignette technique is a method that can detect DIF and adjust for some of these cross-cultural biases that lead to item DIF (King et al., 2004; King and Wand, 2007; Hopkins and King, 2010). Understanding the impact of DIF is important for the development of new assessment tools and especially for their application. The anchoring vignettes provided in this study are a useful and easily applicable technique that can be applied to existing personality measures in cross-cultural research, which will help combat DIF.

To sufficiently support our hypotheses, the introduction is organized as follows. First, we provide an overview of traditional techniques for detecting DIF, followed by a summary of Anchoring Vignettes (*AV*s) and why they are superior over traditional methods. Second, we summarize how *AV*s have been applied both generally and specifically in personality research, specifically in regards to the Big Five Personality factor model. Finally, we demonstrate the utility of *AV*s for combating DIF in the assessment of the Big Five Personality factors, based on data from two countries: Rwanda and the Philippines.

The following passage describes several traditional techniques that are applied to detect DIF. Huang et al. (1997) used the five-factor personality model in two cultural contexts, the Philippines and America, and compared two DIF-statistics to examine measurement equivalence at the item level: (1) Item Response Theory (IRT) and (2) Mantel-Haenszel method (e.g., Ellis et al., 1993; Huang et al., 1997). DIF can be detected by classic IRT statistics, such as item discrimination and item difficulty (e.g., Camilli and Shepard, 1994), or by the area between two cultures' item characteristic curves (Thissen et al., 1988). IRT item parameters are often assumed to be invariant over groups of participants. However, this is often not true (Rupp and Zumbo, 2006). Instead, parameter invariance can be assessed to identify items that lack measurement equivalence, which means the item assesses the central construct differently for each group. However, it is argued that demonstrating factor congruence across cultures does not guarantee measurement equivalence (Bijnen et al., 1986; Huang et al., 1997).

The Mantel-Haenszel, a chi-square statistic comparing the actual and expected frequencies, can be used to detect DIF (Holland and Thayer, 1988). This method has a lot of advantages including its simplicity and easy implementation. However, it does not detect *non-uniform-*DIF, which is an interaction between trait level and group membership so that mean differences in trait level between cultures would not be detected (Rogers and Swaminathan, 1993).

Most historical DIF-statistics focused on binary scored items. Ordinal scaled items, such as Likert-scale items, require a different treatment, such as a logistic regression. Zumbo (1999) estimated a logistic regression to test DIF for ordinal scored items using the responses as a dependent variable with a grouping variable, total scale score, and an interaction of the group and the total scale score as independent variables (e.g., Crane et al., 2006).

DIF can be also detected using multiple-group confirmatory factor analysis through establishing measurement invariance (Thissen et al., 1988; Stark et al., 2006; Teresi, 2006). Configural invariance, the first step in testing measurement invariance, models the same factor structure across groups (Vandenberg and Lance, 2000; Stark et al., 2006). In the case where a sample does not demonstrate configural invariance across countries, it can be assumed that single items, or even the whole test, is affected by DIF (Teresi, 2006). Likewise, DIF can be detected by comparing item factor loadings (e.g., Eysenck et al., 1993).

The use of *AVs* (Thissen et al., 1993) is perhaps the most promising approach that can be applied to detect DIF and the method offers the possibility to correct for it. The idea is to relate self-report answers with external benchmarks that measure the same concept but are more likely to be free of biases and therefore free of some DIF forms. Anchors specifically have been proposed as a useful tool to adjust the answers of different individuals to one underlying standard scale (King et al., 2004). Anchors normally include descriptions (within vignettes) of one hypothetical person who, based on the theoretical description of the trait of interest, is described in a way to illustrate a certain trait level (Chevalier and Fielding, 2011). Each participant then evaluates the behavior of this person on the same scale they used to answer the self-report questions. Because the anchors provide an external benchmark, *AV*s have a number of advantages over traditional DIF-detection procedures (Mõttus et al., 2012). Primarily, while traditional DIF-statistics (described above) essentially plot single item scores against latent trait scores, with both types of scores derived from the same data, with *AV*s, there is independence between the scores (Mõttus et al., 2012).

The following passage provides a short and general overview over the application of *AV*s in different research areas. *AV*s are not widely employed, though there are a few isolated instances of their use. They have been used in work related research (e.g., Kristensen and Johansson, 2008), in research on life satisfaction (e.g., Kapteyn et al., 2010) and quality of life (e.g., Crane et al., 2016), in personality research (e.g, Mõttus et al., 2012), and in an educational context (e.g., student-reported teachers' classroom management; OECD, 2012; Vonkova et al., 2015). These applications mostly indicate that the use of *AV*s was beneficial and resulted in a more valid measure that offered cleaner comparisons between groups. Research on life satisfaction (Angelini et al., 2014), for example, found that Danes and Italians report different levels of life satisfaction. But after adjusting self-report answers with *AV*s, these differences disappeared. Likewise, *AV*s helped improve measurement invariance in the Programme for International Student Assessment *(PISA)* (He and Van de Vijver, 2016), and *AV*s were effectively used to identify and correct for DIF in a self-report physical health scale (Knott et al., 2016).

Personality research, which is conducted in several countries, can especially benefit from the application of *AV*s. A long history of psychological research has shown that the Big Five Factor model of Personality represents the set of constructs that are most strongly differentiated, non-overlapping, and predictive across domains (Roberts et al., 2015). Although they were first discovered in the English language, replication studies in other languages yielded the same five factors (see e.g., McCrae and Terracciano, 2005; Schmitt et al., 2007). But already Allport and Odbert (1936) noticed that culture and time period can influence responses. There are especially large differences in answering personality items when comparing Western and non-Western cultures (e.g., Mpofu and Nyanungo, 1998; Byrne and Campbell, 1999). DIF in personality items is known to appear because of inadequate translation, research, or development, sampling biases, and different response styles (e.g., Grimm and Church, 1999; Van de Vijver and Leung, 2000; Schmitt et al., 2007).

*AV*s have been shown to increase the reliability of scales assessing Conscientiousness and Openness in a representative study of 12th grade students in Brazil (*N* = 8,582) (Primi et al., 2016). The study applied a set of three vignettes for Conscientiousness and Openness. Interestingly, they showed that the Openness vignettes were more frequently misordered relative to the Conscientiousness vignettes. In a study using *AVs* to compare facet-level measures of Conscientiousness across 21 countries, it was determined that, contrary to expectations, self-reported Conscientiousness was minimally affected by cultural differences (Mõttus et al., 2012). Hence, the researchers concluded that it is not necessary to address comparability problems using *AVs* in personality. However, the sample size for each country was relatively small and some of the vignettes were abstract and most likely had differences in meaning due to the numerous translations. Hence, it is possible that the participants applied different standards in answering the *AVs* and the self-report personality questionnaires and hence violated the assumption of Response Consistency (discussed in detail below). Likewise, Primi et al. (2016) as well as Mõttus et al. (2012) do not report whether or not they tested any measurement assumptions (i.e., Response Consistency and Vignette Equivalence, which are described in detail below), which must be fulfilled in order to use *AV*s. He et al. (2017) compared different methods and procedures to improve the comparability between cultures including a vignette set with two levels of conscientiousness (*N* = 3,560 university students in 16 countries). They reported that the vignette sets showed a lack of invariance (arguably due to the characteristics of the vignettes) and hence were not free of DIF. However, they also found that the vignette technique was the only method which resulted in higher internal consistencies. Likewise, the use of *AV*s for the assessment of self-reported teamwork led to increased test information and item discrimination, and higher factor loadings and better model fit in a confirmatory factor analysis (Ham and Roberts, 2015).

In our study, we evaluated the use of *AVs* in the assessment of personality in two countries: Rwanda and the Philippines. We selected these countries for several reasons. First, Rwanda is one the few African countries where the Big Five have not been replicated (Roberts et al., 2015). Thus, the comparison of responses from a country where DIF in the Big Five has already been shown, specifically the Philippines, with another country where the Big Five factor structure have not been replicated, and has a different cultural and historical experience, is informative. Identifying a different structure to the Big Five Factor model for Rwanda would challenge the previously proclaimed generalizability of the Big Five Factor model.

Reviewing cross cultural personality research where *AV*-adjustment was not applied suggests a mean trait-level difference between the Philippines and Rwanda. Researchers who administered the BFI in 56 nations using 28 languages found significant differences in Openness to Experience in the geographical regions of South East Asia compared to other world regions (Schmitt et al., 2007). Likewise, a comparison of the United States and the Philippines on the Big Five found significant mean differences between the groups. However, in that study, almost 40% of the items administered in the Philippines showed DIF, despite surveying both groups in English (Huang et al., 1997).

There is also evidence suggesting a cultural difference in the approach toward a self-report questionnaire and the use of the response options. For example, Rwandans tend to place an extremely high value on authorities and people in a high status position, which can affect responding on self-report questionnaires (Staub et al., 2005). Likewise, citizens of several African countries (i.e., Benin, South Africa, Senegal, and Burkina Faso) and Southeast Asia (e.g., the Philippines) showed the highest rates of extreme responding to self-reported personality (Mõttus et al., 2012). In contrast, Germany shows the lowest rates of extreme responding, and most European nations and the United States are characterized by medium extreme responses. Thus, this suggests that individuals from the Philippines and African countries are characterized by a difference in understanding and interpretation of self-report questionnaire items, namely those assessing personality, which could explain the aforementioned mean trait-level differences. As such, it is important to test the extent to which there is DIF between the Philippines and Rwanda and whether this can be addressed through the use of *AV*s. As our study is the first to apply vignette sets (with three levels) on the BFI-44, comparison to previously published results with other countries is not possible.

The Big Five are linked to several important aspects of our life. Life satisfaction, a component of subjective well-being, is correlated to the Big Five, but the correlations are somewhat inconsistent between countries. In a representative Dutch sample, life satisfaction had small to medium positive correlations with all Big Five factors (Müller, 2014). However, in an Iranian sample, researchers found small negative correlations of life satisfaction with Conscientiousness, Openness, and Extraversion (*r* = −0.24 to −0.28) and non-significant correlations with Neuroticism and Agreeableness (Hosseinkhanzadeh and Taher, 2013). In a Nigerian sample, correlations of life satisfaction with Neuroticism were negative but positive with the other Big Five factors (Onyishi et al., 2012). The Big Five are also linked to work behavior. In a USA sample, all Big Five dimensions had small to medium negative correlations with counterproductive work behavior (Mount et al., 2006). The extent to which this relation differs between cultures is unclear.

Overall, we hypothesize that using the *AV* technique will improve the psychometric characteristics and the cross-cultural comparability of self-reported personality. Here are our specific hypotheses:

*Hypothesis 1*: Using *AVs* to adjust self-report responses will improve the BFI-44 reliability, measured with omega ω (an indicator of factor saturation; McDonald, 1999), for each of the Big Five Personality factors, with estimates ranging from good to excellent. This hypothesis will be tested by comparing the overlap of the 95% confidence intervals of the omegas.
*Hypothesis 2: AV*-adjusted scores fitted in a graded response model will show an increase in discriminant power, relative to the original scores, resulting in a wider range of thresholds and larger discrimination parameters. Furthermore, the increase in overall test information for the *AV*-adjusted scores will be indicated by a broader range of θ-levels and small standard errors.
*Hypothesis 3:* In a confirmatory factor analysis, models of the Big Five based on *AV*-adjusted scores will show acceptable fit to the data (CFI ≥ 0.90 and RMSEA ≤ 0.08) and a correlational structure such that Neuroticism is weakly negatively correlated with all of the other dimensions, and the other dimensions are weakly positively correlated with each other, supporting the theoretical structure of the Big Five model. We expect this result to hold for both samples.
*Hypothesis 4:* Test-criterion relationships of life satisfaction and counterproductive work behavior with the *AV*-adjusted Big Five factor scores will be significantly stronger for the *AV*-adjusted scores.

## Methods

### Procedure and sample

The studies were conducted in Rwanda (Sample 1) and in the Philippines (Sample 2). Participants were recruited through the educational institute Akilah Institute for Woman of Akazi College in Africa, which is partially supported by the Educational Development Center (*EDC*) in Washington D.C. This study was carried out in accordance with the recommendations of the institutional review board *(IRB*; *IRB* Registration: IRB00000865) of the *EDC* Human Protections department. In addition, all participants provided written informed consent in accordance with the Declaration of Helsinki. In the Philippines, the parents of the participants were also informed of the study and the possible involvement of their child with a letter.

The newly-developed *AV*s were tested in psychology labs before the study was conducted. All items and translations were reviewed several times by all institutes involved. The studies were conducted by local interviewers who were employed and trained for data collection by the *EDC*. Each participant completed the questions in the same order, which were presented on a tablet provided by the *EDC*. Due to technical problems and power supply issues in both countries, some participants completed paper-pencil versions of the test material. The paper-pencil versions were entered into an electronic database by the local staff. Participation in these studies was voluntary and participants could withdraw from the study at any moment. The samples are summarized in Table 1. The samples are based on adolescents and young adults who were either finishing school and/or applying for jobs. Several articles support this application of the Big Five in young adult and adolescent samples (see for example, Bratko and Marušić, 1997; Digman, 1997; Ehrler et al., 1999; Rothbart et al., 2000). This sampling procedure resulted in a relatively homogeneous sample regarding age and education, which were biases what we wanted to avoid. The participants completed the study during their college course time; therefore, they did not receive any financial compensation.

**Table 1:** Descriptions of the Rwanda and Philippines samples.

	***N***	**Age**	**Sex**
Sample 1: Rwanda	423	*M* = 21.79 *SD* = 2.7	Female: *N* = 356 (84%)
		Range: 15–33	Male: *N* = 67 (16%)
Sample 2:Philippines	143	*M* = 15.5 *SD* = 0.83	Female: *N* = 99 (69%)
		Range: 14–19	Male: *N* = 44 (31%)

*The Rwanda sample includes two different schools in different regions of Rwanda. The Philippines sample includes one school*.

### Measures

All measures were translated from English into either Kinyarwanda or Filipino. The translation was supervised by the *EDC* using backward and forward translation checks. The participants completed demographic questions first where they were asked to provide information about themselves, their family, and their home situation. These questions were also tailored for each country; for example, Filipinos were asked if they have a computer at home and Rwandans were asked if they have access to running water. Therefore, the demographics differed between both samples. As the studies were part of a larger project, additional measures were also included including 10 Situational Judgment Tests for Conscientiousness and the Conscientiousness Facet-Tool (MacCann et al., 2009). Because of the focus of the article, these measures are not discussed further.

#### Anchoring vignettes

The *AVs* included 15 hypothetical descriptions; three hypothetical descriptions for each personality dimension of males or females who embodied a certain level of the corresponding personality dimension. The *AVs* were developed by scientists from the Professional Examination Service in New York. Table 2 shows *AVs* for the Big Five, which show different levels of Conscientiousness, Agreeableness, Neuroticism, Openness, and Extraversion. In the first *AV* for conscientiousness, Sophia represents someone with a low level of Conscientiousness. In the second, Jacob shows a medium level of Conscientiousness, and in the third, Emma displays a high level of Conscientiousness. Participants were asked to rate the extent to which they agreed with the statement that Sophia, Jacob, and Emma are Conscientious. In this case, the suggested ratings for following the correct order would be “disagree strongly” or “disagree a little” for Sophia's statement, “neither agree nor disagree” for Jacob's statement, and “agree a little” or “agree strongly” for Emma's statement. Therefore, the person in Vignette 1 is rated as having lower Conscientiousness relative to the person in Vignette 2. Also, the person in Vignette 3 is rated as having the highest conscientiousness and is therefore higher than on conscientiousness the persons in described in Vignette 2 and 1.

**Table 2:** *AV*s for Conscientiousness (C), Agreeableness (A), Neuroticism (N), Openness (O), and Extraversion (E).

**How much do you agree with this statement?**	**Disagree strongly**	**Disagree a little**	**Neither agree nor disagree**	**Agree a little**	**Agree strongly**
C1. Sophia tends to be somewhat careless. Other workers also comment that she is lazy. Sophia often also appears disorganized. Based on this information, to what extent do you agree with the statement “Sophia is conscientious/hard-working”?	O	O	O	O	O
C2. Jacob is a reliable worker and does all work with great efficiency, but he is easily distracted. Based on this information, to what extent do you agree with the statement “Jacob is conscientious/hard-working”?	O	O	O	O	O
C3. Emma always does a thorough job. She perseveres until all tasks are finished. Emma also makes plans and follows through with them. Based on this information, to what extent do you agree with the statement “Emma is conscientious/hard-working”?	O	O	O	O	O
A1. Jean tends to disagree with others, and as a result often starts quarrels. Indeed, many people consider Jean quite rude. Based on this information, to what extent do you agree with the statement “Jean is an agreeable person”?	O	O	O	O	O
A2. Even though Nicole is helpful and unselfish with others, some people find her cold and unfriendly. This does not matter so much, as she has a forgiving nature. Based on this information, to what extent do you agree with the statement “Nicole is an agreeable person”?	O	O	O	O	O
A3. Claude is considerate and kind to almost everyone. He is very trusting, and finds it easy to cooperate with others. Based on this information, to what extent do you agree with the statement “Claude is an agreeable person”?	O	O	O	O	O
N1. Carine frequently appears quite depressed to other people. She gets nervous easily. Based on this information, to what extent do you agree with the statement “Carine is emotionally stable”?	O	O	O	O	O
N2. Although in tense situations Paul remains calm, he can be quite moody. And he tends to worry quite a lot. Based on this information, to what extent do you agree with the statement “Paul is emotionally stable”?	O	O	O	O	O
N3. Aline always appears relaxed and to handle stress well. Indeed, she never comes across as upset. Aline remains calm in all situations. Based on this information, to what extent do you agree with the statement “Aline is emotionally stable”?	O	O	O	O	O
O1. Emmanuel has few artistic interests, and is not especially sophisticated either in music or literature. This has led some people to observe that Emmanuel does not appear especially curious about anything. Based on this information, to what extent do you agree with the statement “Emmanuel is open-minded”?	O	O	O	O	O
O2. Emma has an active imagination. This has led some people to calling her a deep thinker. Even so Emma prefers work that is routine. Based on this information, to what extent do you agree with the statement “Emma is open-minded”?	O	O	O	O	O
O3. Jean Bosco is original and always coming up with new ideas. This has led some people to calling him inventive. But beyond this, Jean Bosco values artistic, aesthetic experiences. Based on this information, to what extent do you agree with the statement “Jean Bosco is open-minded”?	O	O	O	O	O
E1. Claudine is very reserved. She tends to be quiet no matter what the circumstance. Indeed, people find her shy and inhibited. Based on this information, to what extent do you agree with the statement “Claudine is extraverted”?	O	O	O	O	O
E2. Emile is often talkative and generates a lot of enthusiasm in others. But on his day, Emile can be rather shy and inhibited. Based on this information, to what extent do you agree with the statement “Emile is extraverted”?	O	O	O	O	O
E3. Rosette has an assertive personality, and as a result appears outgoing and sociable. Indeed, people are always commenting on how full of energy Rosette is. Based on this information, to what extent do you agree with the statement “Rosette is extraverted”?	O	O	O	O	O

#### The big five inventory (BFI-44)

The BFI-44 (John et al., 1991; Benet-Martínez and John, 1998) uses 44 items to measure the Big Five Personality factors: Extraversion (e.g., “*I am someone who is talkative”)*, Agreeableness (e.g., “*I am someone who is helpful and unselfish with others”)*, Conscientiousness (e.g., “*I am someone who does a thorough job”)*, Neuroticism (e.g., “*I am someone who is depressed, blue”)*, and Openness (e.g., “*I am someone who is original, comes up with new ideas”)*. The items are answered on a five-point Likert-scale with the poles “disagree strongly” and “agree strongly”. John and Srivastava (1999) established the validity and factor structure of this measurement. The reliabilities before and after the *AV*-adjustment are presented in the results section.

#### Satisfaction with life scale

This scale measures global life satisfaction with five items (e.g., “*I am satisfied with my life”*). It is known for good internal reliability and validity (Diener et al., 1985). The reliability of the scale for the whole study was acceptable (ω = 0.76).

#### Counterproductive behavior

This construct was measured with an adaption of the Interpersonal and Organizational Deviance items (Bennett and Robinson, 2000) for a school and work context (e.g., “*How often have you publicly embarrassed someone at school or work”*). Respondents answered the items on a seven-point Likert-scale ranging from “never” to “daily.” The original instrument shows an acceptable fit in a confirmatory factor analysis and a two-factor structure. Our shorter adapted form has acceptable reliability (ω = 0.79).

### Statistical analysis

#### Data cleaning

To appropriately test the hypotheses and address cross-cultural comparability, we took several steps in terms of data cleaning and scoring prior to calculating the models. For data cleaning in both studies, we applied the following *a priori* standards to decrease noise in the data. Noise in the data can be due to inattentive participants or participants that are not willing to or fail to follow instructions (cf. Oppenheimer et al., 2009; Maniaci and Rogge, 2014). Noise can lead to low variance or indicate inappropriate response patterns in the data (e.g., participants always selecting the same response option), and it can lead to consistent order violations of the *AVs* due to the inattentive reading of instructions. Therefore, we removed participants with:

1. more than 10% missing entries in the data
2. low variance (<0.5) in answering the self-report questionnaires and *AVs*
3. inappropriate response patterns in the *AVs*
4. consistent order violations in the *AVs*

18.7% of the original *N* = 520 participants in Sample 1 (Rwanda) and 28.5% of the original *N* = 200 participants in Sample 2 (Philippines) were removed in accordance to these criteria. Most participants were removed because of omissions in the data, which mostly originated from the paper-pencil versions.

### Analyzing the anchoring vignettes

In our study, we used a set of three vignettes varying in intensity to adjust self-report responses using a non-parametric approach. Specifically, responses to the self-report questionnaires were compared against the responses to the vignettes (see Table 2 for all examples relating a single self-report answer to a set of three vignettes). In this process, the responses of the original 5-point Likert-scales spread across a new 7-point Likert-scale. These adjusted answers are hypothetically free of some DIF forms and can thus be analyzed and interpreted like any other Likert-scale (King et al., 2004; Wand, 2013).

Table 3 shows the *AV*-adjusted scores if the participant rates the vignettes in the defined order. Of course, participants show individual differences in their ratings of these vignettes. For example, it is possible for participants to evaluate two vignettes equally if they decide that two hypothetical persons display the same intensity of a trait. Hence, they do not distinguish between two or even three vignettes (e.g., Vignette 1 = Vignette 2 < Vignette 3), which is referred to as tying one or more vignette pairs. Alternatively, participants can rate the vignettes as having a different intensity than originally defined, such as rating Vignette 2 as lower than Vignette 1 (Vignette 2 < Vignette 1 < Vignette 3) when the correct order is Vignette 1 < Vignette 2 < Vignette 3.

**Table 3:** Rules for recoding self-report responses with three *AVs*.

**Relative order ratings**	**Adjusted score**
Self < Vignette 1 < Vignette 2 < Vignette 3	1
Self = Vignette 1 < Vignette 2 < Vignette 3	2
Vignette 1 < Self < Vignette 2 < Vignette 3	3
Vignette 1 < Self = Vignette 2 < Vignette 3	4
Vignette 1 < Vignette 2 < Self < Vignette 3	5
Vignette 1 < Vignette 2 < Self = Vignette 3	6
Vignette 1 < Vignette 2 < Vignette 3 < Self	7

“*Self” represents a single self-report answer about one's own personal traits. Vignettes 1, 2, and 3 are the corresponding vignette set measuring the same trait, with the trait level highest in Vignette 3, followed by Vignette 2, with the trait level lowest in Vignette 1*.

These ties and order violations add complexity to analyses resulting in fragmentary information (Hopkins and King, 2010). If the participant orders the vignettes in the correct order, the non-parametric adjustment through *AV*s eventuate in a single value (see Table 2). Ties and order violations instead result in an interval solution and therefore in a set of values (King et al., 2004). In our example, the interval can range from one to seven. The non-parametric approach has only a limited range of options for dealing with ties and order violations in *AV*s (Paccagnella, 2013). Previous research has shown that choosing the lower bound of these intervals as an *AV*-adjusted answer leads to improved reliabilities (Kyllonen and Bertling, 2014).

It is important that the assumptions of vignette equivalence and response consistency are met before evaluating *AV*-adjusted scores (King et al., 2004). *Vignette equivalence* means that every participant perceives the *AV*s in the same way and therefore with the same ranking (Grol-Prokopczyk et al., 2015), which should generally occur. This assumption would be violated if a large proportion of participants systematically interpret the vignettes in another way. Violation of this assumption leads to problems in non-parametric adjustments and incomparable thresholds in the parametric approach. In previous literature, this assumption was either assumed prima facie (Grol-Prokopczyk et al., 2015) or considered to be met by simply looking at the consistencies when rank-ordering the vignettes (King et al., 2004; Kristensen and Johansson, 2008; Rice et al., 2011). However, this assumption can be assessed by analyzing the amount of order violations within the *AV*s rank-order, with 10% or more indicating a significant amount of order violations. Generally, order violations are treated as measurement error. However, patterns in order violations can also have a diagnostic impact, providing information about the sample, the translation, or the quality of the vignette. For example, the World Health Organization (WHO) self-care vignettes show an order violation of 35.71% compared to an average 10% order violation for the other WHO vignettes. Systematic order violations can be also due to isolated “bad vignettes” (Grol-Prokopczyk et al., 2015). Only if there are patterns in order violations is it problematic to analyze *AV*s based on the non-parametric approach (described later).

The assumption of *response consistency* tests the extent to which participants use the same thresholds for answering the self-report items and the *AV*s. Response consistency is violated if participants apply alternative standards to the self-report items and to the *AVs* or use varying standards in answering the *AV*s. Violations of this assumption lead to problems in adjusting self-report responses with the non-parametric approach (Grol-Prokopczyk et al., 2015). There are options to test response consistency, such as comparing the thresholds of the *AV*s and the self-report items collected in different waves (Kapteyn et al., 2011) or comparing thresholds of objective measures and self-report measures with responses to the *AV*s (Gupta et al., 2010; Soest et al., 2011; Hirve et al., 2013). However, these options are often not available. Instead, another possibility is simply examining the IRT parameters, specifically the overlap of the threshold confidence intervals in a graded response model (i.e., a mathematical model for ordered polytomous categories) for the *AVs*. Mostly, this assumption is not examined but is instead assessed indirectly through interpreting the plausibility of the study results (King et al., 2004; Grol-Prokopczyk, 2014).

**Figure 1:**
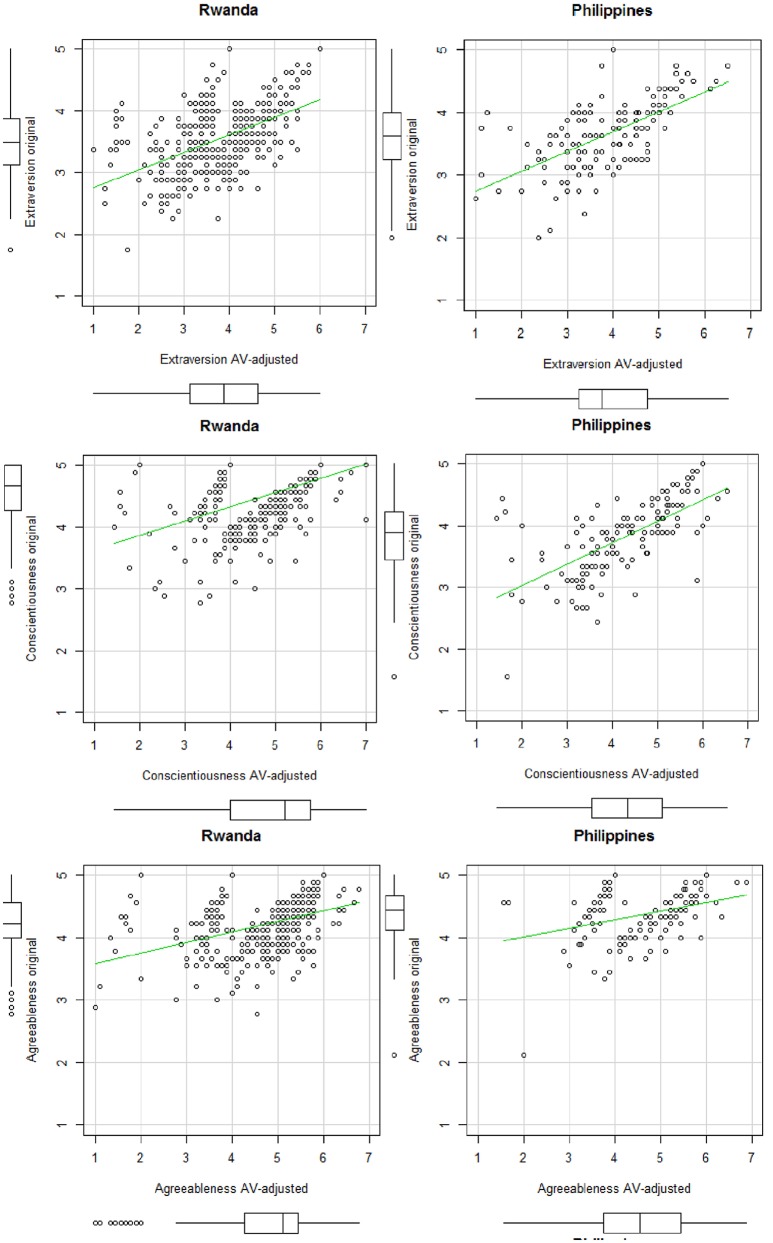
Scatterplots of the original and the *AV*-adjusted scales for Rwanda (*N* = 423) and the Philippines (*N* = 143).

**Figure 2:**
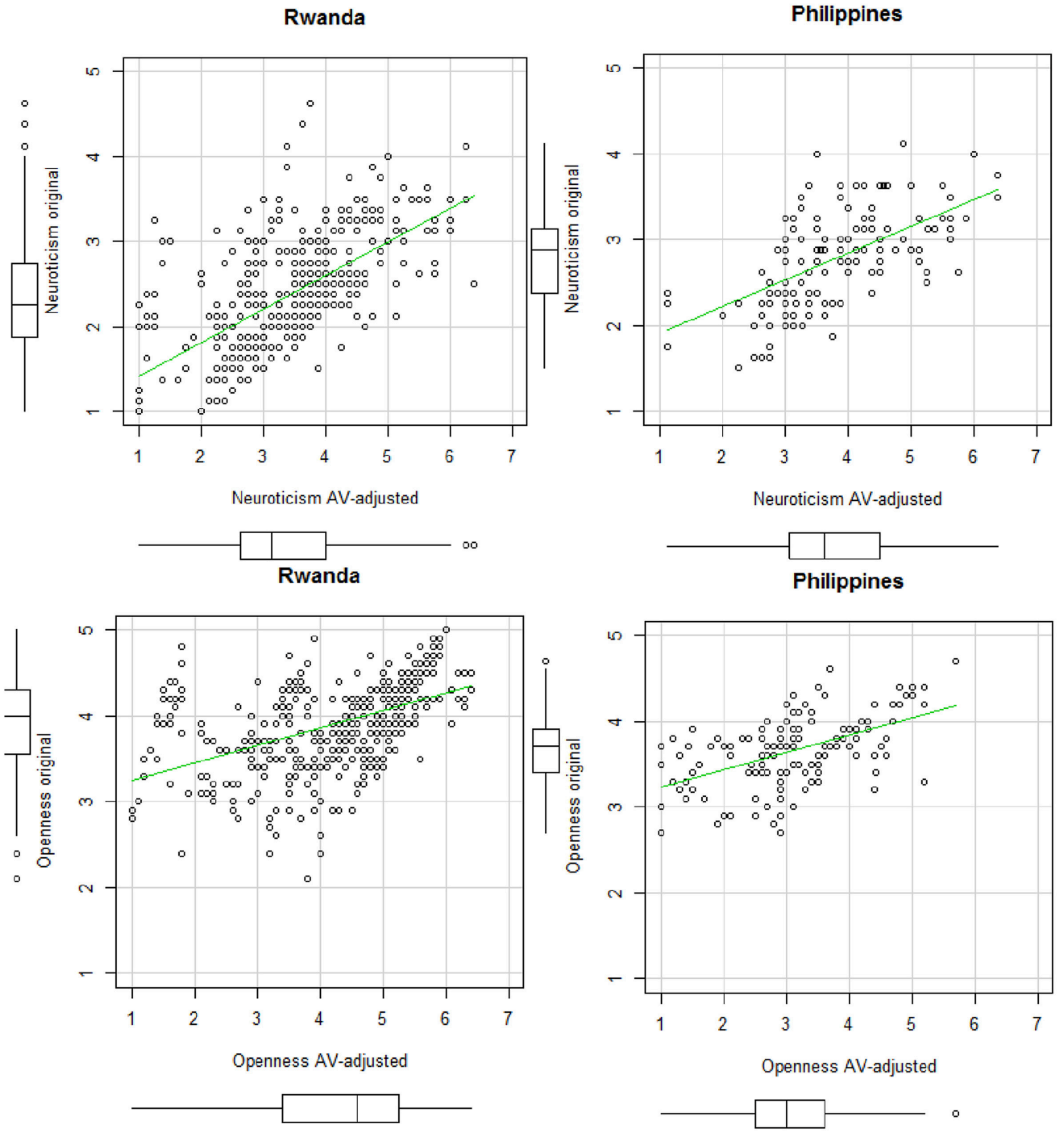
Same as Figure 1.

In our study, *AVs* were analyzed with the *anchors* package in R Studio version 3.3.2 (Wand et al., 2011). Using this package, we assessed entropy (King and Wand, 2007), which is an indicator of the informativeness of a given *AV* set. These statistics showed that all vignette sets, including the three vignettes in their defined order, were mostly informative. Next, we applied the non-parametric approach and calculated the *AV*-adjusted scores. Table S1 in Supplemental Material shows an example of R-Code syntax used for the analysis of the *AV*s. Based on recommendations in the literature, we treated order violations as ties and chose the lower bound of the intervals (Kyllonen and Bertling, 2014). Figures 1, 2 show the means for the BFI-44 items for the original 5-point Likert-scale before the *AV*-adjustment and the means of the 7-point Likert-scale after the *AV*-adjustment, separated by country.

### Computational approach

We assessed DIF through a multiple-group confirmatory factor analysis, establishing configural measurement invariance on the Big Five factor model between both samples. Then, several indices and programs were used to evaluate support for our hypotheses.

The first hypothesis was tested by applying McDonald's ω (McDonald, 1999), an estimate of general factor saturation that is considered a better indicator of reliability than Cronbach's α (Zinbarg et al., 2005), and the confidence intervals of ω were interpreted to evaluate whether reliability significantly improved after AV-adjustment.

For testing Hypothesis 2, we used a graded response model for the original and the *AV*-adjusted scores. The graded response model belongs to the polytomous item response theory models and can be applied for ordinal manifest variables. Reise and Waller (1990) showed that two-parameter logistic *IRT* models can be used for multidimensional data, which describes Personality questionnaire data. The graded response model was fitted with the multidimensional item response theory (full-information item factor analysis) *MIRT* package in R Studio version 3.3.2 (Chalmers, 2012).

DIF and Hypothesis 3 were assessed based on a confirmatory factor analysis with the BFI items as manifest indicators of their respective Big Five Personality factor, which were allowed to correlate. Previous studies have reported problems in modeling personality self-report questionnaires in a confirmatory factor analysis (Marsh et al., 2006). For example, the NEO-PI-R is known for encountering problems such as model misfit, negative item loadings, and high error correlations (e.g., Borkenau and Ostendorf, 1990; McCrae et al., 1996). A common technique for improving model fit in such a situation involves deleting items that are not loading high enough onto the corresponding factor (Aluja et al., 2006; Tully et al., 2011). Based on this procedure, items that were completely misfitting the model through small or even negative loadings were deleted from the original measure. In this study, this procedure resulted in a 36-item version of the BFI. To ensure comparability, all models are based on a 36-item solution. In the evaluation of Hypothesis 3, we modeled the 36-item solution of the BFI-44 for the unadjusted self-report scores [Model #1 (Rwanda) and #3 (Philippines)] and for the *AV*-adjusted scores [Model #2 (Rwanda) and #4 (Philippines)]. We then compared improvement in fit for each country (Model #1 vs. Model #2 and Model #3 vs. Model #4). A final model (Model #5), which includes all participants and is based on the *AV*-adjusted scores, was estimated to evaluate whether the *AV*-adjusted scores provide stronger support for the Five Factor structure (i.e., orthogonal factorially-pure scales). As all models are based on the same factor structure based on the same 36 items, models can be compared by looking at improvement in the fit indices.

**Figure 3:**
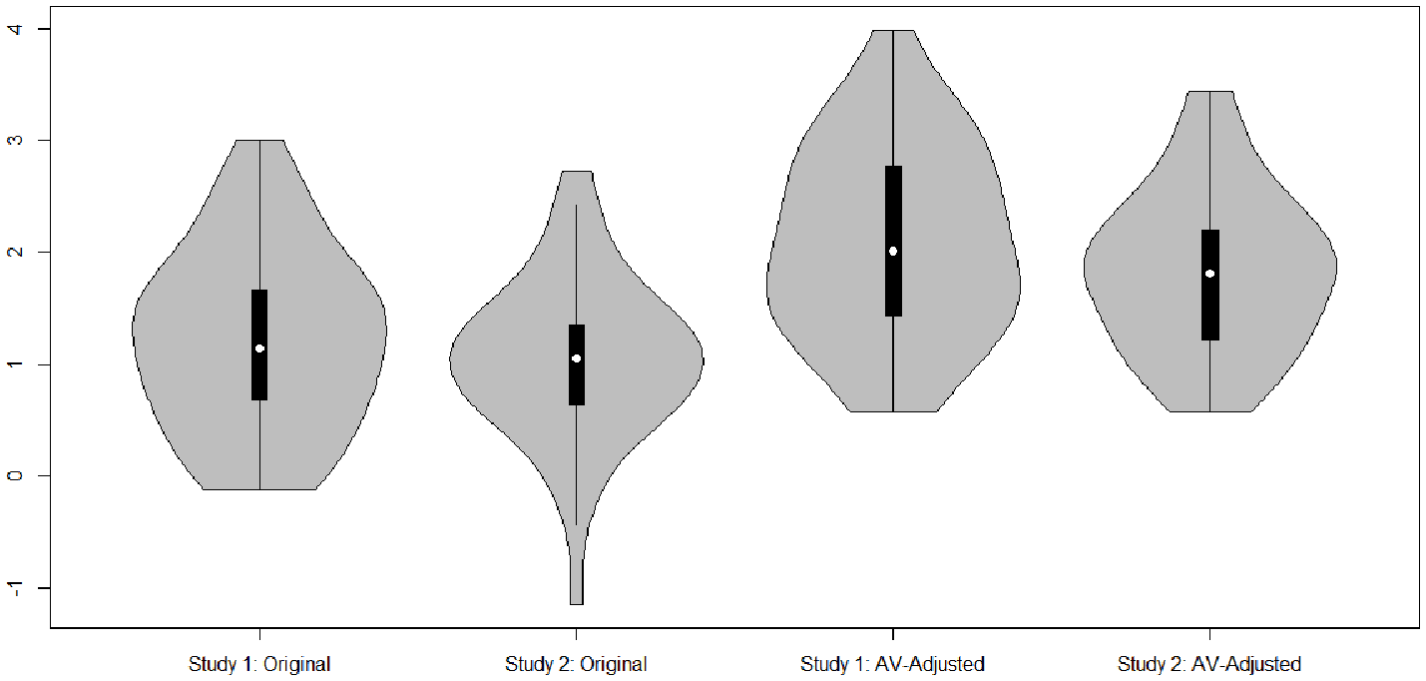
Violin plots of discrimination parameters of the original self-report questions and the *AV*-adjusted answers for Rwanda and Philippines.

In a confirmatory factor analysis, several indices can be used to describe the fit between the theoretical model and the actual model. We used the criteria that a Comparative-Fit-Index (CFI) (Bentler, 1990) greater than or equal to 0.90 and a Root Mean Square Error of Approximation (RMSEA) (Steiger, 1990) less than or equal to 0.08 indicates acceptable fit (Steiger, 1990). The confirmatory factor analyses were conducted with the either Mplus 7 (Muthén and Muthén, 1998-2015) or the *lavaan* package in R Studio version 3.3.2 (Rosseel, 2012). Basic statistics are based on the *psych* package (Revelle, 2014) and the violin plots in Figure 3 are based on the package *vioplot* (Adler, 2005).

## Results

### Evaluating DIF

First, we tested the extent to which the original scores were affected by DIF. To this end, we conducted a multiple-group confirmatory factor analysis, with the above described fit standards for fit indices. In the first model, with all 44 items, imposing configural measurement invariance led to poor model fit (*CFI* = 0.60, *RMSEA* = 0.06). This indicates that the underlying five-factor model is not comparable between samples, and different constructs are represented. Likewise, there were large differences between the factor loadings, which ranged from −0.55 to 0.78. Based on these results, we conclude that configural invariance between both studies on the full scale is not met and hence different constructs are assessed.

To compare improvement in fit from the original scores to the *AV*-adjusted scores, we conducted a second multiple-group confirmatory factor analysis based on the original self-report scores, but with the 36-item solution (Baseline Model: *CFI* = 0.67, *RMSEA* = 0.07). The poor model fit supports our initial conclusion that the original self-report items are affected by DIF and need an *AV*-adjustment to achieve cross-cultural comparability.

### Vignette equivalence and response consistency

Next, before interpreting the AV-adjusted scores, we checked vignette equivalence and response consistency assumptions. The percentage of vignettes with the correct order ranged from 26 to 66% (Rwanda) and 37 to 72% (Philippines; see Table 4 for the percentage of all possible orders for each domain). In our study, the chance of randomly violating the correct order is 74% while the chance of randomly correctly ordering the *AV*s is 8%. Violations of 10% or lower can be treated as measurement error, however systematic violations (e.g., participants using answer pattern resulting in order violations) have to be excluded. Conscientiousness, Agreeableness, and Extraversion for both samples, as well as Openness for the Philippines sample, had order violations of 10% or less, indicating no problematic vignettes. However, violations for Neuroticism (Rwanda: 15%; Philippines: 18%) and Openness for the Rwanda sample (23%) were higher. For the latter, Openness displayed a higher amount of ties (51%) than correct orders (26%), in addition to the large percentage of order violations. Given there was only a partial violation of the vignette equivalence assumption, we felt comfortable continuing in the analyses. Possible reasons for these violations, as well as examples of other research where the same violations were found, is discussed in the Discussion section.

**Table 4:** Percentage of correctly ordered vignettes, vignette ties, and order violations for each Big Five Personality factor, and the percentage of random chance ordering.

	**Correct order (1 < 2 < 3) random chance of correct order = 8%**	**Ties (e.g., 1 = 2 < 3) random chance of ties = 18%**	**Order violations (e.g., 2 < 1 < 3) random chance of order violation = 74%**
**CONSCIENTIOUSNESS**			
Rwanda	66%	30%	4%
Philippines	72%	18%	10%
**AGREEABLENESS**			
Rwanda	61%	33%	6%
Philippines	52%	43%	5%
**NEUROTICISM**			
Rwanda	42%	43%	15%
Philippines	37%	45%	18%
**OPENNESS**			
Rwanda	26%	51%	23%
Philippines	49%	41%	10%
**EXTRAVERSION**			
Rwanda	47%	44%	8%
Philippines	56%	34%	10%

Response consistency was evaluated through an examination of whether or not respondents used the same thresholds while answering the *AVs* and the self-report questionnaire. We fitted a graded response model for the *AVs* and the self-report questions and compared the four thresholds of the *AVs* against the corresponding self-reports. The confidence intervals of most thresholds overlapped. Therefore, we consider the Response Consistency requirement met.

### Hypothesis testing

Next, we applied the non-parametric approach (King et al., 2004) on the data to produce *AV*-adjusted scores. These *AV*-adjusted scores are what was compared with the original self-report answers.

*Hypothesis 1:* Reliability before and after AV-adjustment

We compared McDonald's ω (McDonald, 1999), a measure of reliability, for the original and *AV*-adjusted scores (see Table 5). For the original scores, omega indicated poor reliability (ω = 0.32 −0.66) for all dimensions except conscientiousness (ω = 0.74). Following the *AV*-adjustment, the omegas increased for every dimension in both samples to acceptable or good (ω = 0.80 −0.92). Looking at the 95% confidence interval, we see that the intervals of the omega estimates for original and the *AV*-adjusted scores do not overlap. In sum, the analysis shows that the *AV*-adjusted scales show better reliability than the original self-report scales, supporting hypothesis 1.

**Table 5:** Scale reliability, indicated by McDonald's omega, and 95% Confidence Intervals for the original and *AV*-adjusted Big Five Personality factors.

	**Rwanda**		**Philippines**	
	**Original**	***AV*-adjusted**	**Original**	***AV*-adjusted**
Conscientiousness	0.74 [0.69 −0.78]	0.92 [0.90 −0.93]	0.76 [0.64 −0.83]	0.89 [0.84 −0.92]
Agreeableness	0.32 [0.21 −0.42]	0.87 [0.83 −0.90]	0.63 [0.45 −0.80]	0.89 [0.85 −0.92]
Neuroticism	0.66 [0.60 −0.72]	0.80 [0.76 −0.83]	0.63 [0.50 −0.70]	0.82 [0.77 −0.87]
Openness	0.66 [0.61 −0.71]	0.91 [0.89 −0.92]	0.57 [0.46 −0.66]	0.88 [0.84 −0.91]
Extraversion	0.51 [0.39 −0.59]	0.81 [0.77 −0.84]	0.62 [0.48 −0.71]	0.82 [0.77 −0.88]

*McDonald's omega and 95% confidence interval of omega for a model with five correlated factors. Original, original (5-point) self-report Likert-scale (before the AV-adjustment); AV-adjusted, 7-point Likert-scale (DIF-free, after the AV-adjustment)*.

*Hypothesis 2:* Item functioning before and after AV-adjustment

Next, we investigated the item and category function of the original and the *AV*-adjusted scores. Here, we assume that the DIF-free scores increase the discriminant power, as compared to the original self-report scores, because they are comparable between countries and more relevant to the measured trait in both studies. The violin plot in Figure 3 shows that, for both studies, the discrimination parameters mostly increase as a result of the *AV*-adjustment.

The discriminant power also increases as the range of the threshold widens. The original self-report scores are based on a 5-point Likert-scale while the *AV*-adjusted scores use a 7-point Likert-scale. This results in a different number of thresholds: b_1_ to b_4_ (original 5-point Likert-scale) and b_1_ to b_6_ (7-point Likert-scale for the *AV*-adjusted answers). As b_1_ to b_6_ span a wider range of values, this indicates that the *AV*-adjusted scores differentiate higher levels of proficiency compared to the original self-report scores. In particular, for the original self-report scores, the last threshold was more effective in differentiation relative to the last category (b_4_). As we compared four to six thresholds, we examined correlations between the thresholds of the original and the *AV*-adjusted scores. This comparison shows that most thresholds are highly correlated with one another (*r*s = 0.47 to 0.94). Only the last threshold of the *AV*-adjusted scores (b_6_) shows negative correlations with the thresholds of the original self-report scores.

Next, we evaluated the overall test information (i.e., the degree of certainty of the proficiency estimates) for the original and *AV*-adjusted scores. The test information curve includes θ-levels from −6 to 6. For the original self-report scores, the test is mostly informative for θ < 0. Above zero, however, the standard error increases greatly and the test information decreases. For the *AV*-adjusted scores, the test is less informative for θ < 0, but it is still informative for θ > 0, particularly between 2 and 4.

Overall, we conclude that hypothesis 2 was supported.

*Hypothesis 3:* Big five factor structure before and after AV-adjustment

Next, we tested our hypothesis that the *AV*-adjusted scores will have improved model fit and a better loading pattern in a confirmatory factor analysis, relative to the original scores, thus showing better support for the Big Five factor structure.

Table 6 shows the results of the confirmatory factor analysis, based on the 36-item version of the BFI. As expected, the models based on the original self-report scores [Model #1 (Rwanda) and #3(Philippines)] have poor fit to the data, yielding a non-positive definite covariance matrix, with item loadings weak in magnitude, not significant, or even negative. These models are not clearly identified but are displayed here for purposes of comparison. In sum, Model #1 and #3 do not support the Big Five factor structure.

**Table 6:** Confirmatory Factor Analysis model fit estimates based on the original and *AV*-adjusted scores.

**#**	**Model type**	**CFI**	**RMSEA**	**χ^2^**
1	Rwanda: Original^+^	0.68	0.06	χ_2_(584) = 1,479
2	Rwanda: *AV*-adjusted	0.90	0.06	χ^2^_(584)_ = 1,423
3	Philippines: Original^+^	0.56	0.08	χ^2^_(584)_ = 1,165
4	Philippines: *AV*-adjusted	0.80	0.08	χ^2^_(584)_ = 1,034
5	Rwanda and Philippines Combined: *AV*-adjusted	0.90	0.05	χ^2^_(584)_ = 1,640

*All models are with the 36-item version. + These models have a non-positive definite covariance matrix, indicating they are not clearly identified; they are displayed her merely for comparison purposes*.

The models including the *AV*-adjusted scores (Model #2, #4, and #5) show high item loadings and better model fit then Models #1 and #3. However, while Model #2 (Rwanda) and #5 (Rwanda and Philippines combined) show acceptable fit, the fit of Model #4 (Philippines) is not acceptable. For Model #5, the model fit improved over the original Baseline Model reported above, which was based on the original scores (*CFI* = 0.67, *RMSEA* = 0.07). Therefore, we assume that the studies are now comparable. In Model #5, Neuroticism is negatively correlated with every other dimension (*r* = −0.17 to −0.31) and all other dimensions are weakly and positively correlated with one another (*r* = 0.13 to 0.31). Thus, the correlations of the *AV*-adjusted scores are much more in line with previous findings than the correlations for the original self-report scores. In sum, the confirmatory factor analysis shows that the *AV*-adjusted scores better support the original factor structure of the Big Five, supporting hypothesis 3.

*Hypothesis 4:* Test-criterion relationships

Finally, we evaluated test-criterion relations with external outcome variables: satisfaction with life and counterproductive behavior (either at school or at work). The outcome variables were correlated with the Big Five Personality factors before and after the *AV*-adjustment (see Table 7). The correlations of counterproductive behavior with Conscientiousness, Agreeableness and Neuroticism, decreased significantly after the *AV*-adjustment. However, for life satisfaction, with two exceptions, correlations were not significantly different after the *AV*-adjustment. The two exceptions are for the Rwanda sample where life satisfaction is negatively correlated to Openness and Conscientiousness after AV-adjustment, but unrelated before the adjustment. Overall, hypothesis 4, which stated correlations with satisfaction with life and counterproductive behavior should be stronger after *AV*-adjustment, was not supported.

**Table 7:** Correlations (Spearman rho) of the Big Five Personality factors, based on the original and *AV*-adjusted scores, with two outcome variables.

	**Life satisfaction**	**Counterproductive behavior**
**CONSCIENTIOUSNESS**		
Rwanda: Original/*AV*-adjusted	−0.01/−0.12^*^	−0.25^**^/−0.19^**^
Philippines: Original/*AV*-adjusted	0.07/0.05	−0.51^**^/−0.30^**^
**AGREEABLENESS**		
Rwanda: Original/*AV*-adjusted	−0.03/−0.04	0.22^**^/−0.13^**^
Philippines: Original/*AV*-adjusted	0.04/−0.02	−0.50^**^/−0.19^*^
**NEUROTICISM**		
Rwanda: Original/*AV*-adjusted	−0.01/−0.01	0.11^*^/0.07
Philippines: Original/*AV*-adjusted	0.08/0.07	0.24^*^/0.12
**OPENNESS**		
Rwanda: Original/*AV*-adjusted	−0.04/−0.14^*^	−0.10^*^/−0.12^*^
Philippines: Original/*AV*-adjusted	0.07/−0.14	−0.05/0.05
**EXTRAVERSION**		
Rwanda: Original/*AV*-adjusted	0.03/−0.05	0.01/0.01
Philippines: Original/*AV*-adjusted	0.15/0.13	−0.02/0.03

** *p < 0.01*;

* *p < 0.05*.

*Correlations presented in italics mean there is a significant difference in relations with that personality factor depending on whether the original or AV-adjusted scoring was used*.

## Discussion

Overall, the results suggest that the *AV* methodology is an appropriate tool for cross-cultural research, although the change in test-criterion relationships warrants further investigation.

### Summary and interpretation of the results

We demonstrated that not even the weakest degree of measurement invariance, configural invariance, was present across both countries. Hence, we show that the BFI-44 test and its items are affected by cross-cultural DIF. Because we performed several backward and forward translation checks, we presume our items showed no evidence of any translation problems, and that the observed DIF most likely occurred because participants from different countries displayed different probabilities of item endorsement. Thus, we utilized *AVs* to correct for DIF.

Before interpreting the AV-adjusted scores, we showed that the *AV*s mostly met the two basic assumptions of vignette equivalence and response consistency. On average, 89% of the participants ordered all vignettes correctly or rated them as ties. Thus, we can assume there was generally vignette equivalence, with most participants perceiving the vignettes in the same way and in the defined order. However, Neuroticism and Openness showed a non-negligible amount of order violations. A possible reason for the order violation on the Neuroticism *AVs* is that they were presented on a reversed scale. In previous studies, the use of a reversed scale also created confusion for participants (He et al., 2017). The order violations within Openness are probably due to the conceptualization of the Openness factor and the corresponding *AV*-set. Primi et al. (2016) discovered similar results and suggested that this is because Openness is different from the other domains and is not as easy to rate as it mostly includes not observable behaviors compared to the other domains, such as Conscientiousness, which has observable behaviors. Likewise, given response consistency is traditionally difficult to confirm or disconfirm, we proposed a novel statistical solution and tested it with the present data set. We showed that the confidence intervals of most thresholds overlapped, implying that participants applied the same thresholds in answering *AVs* and the original self-report questions. Thus, we could conclude that the response consistency assumption was met.

We examined how the *AV*s influenced other psychometric characteristics by testing a series of hypotheses. In evaluation of the first hypothesis, we examined scale reliability, estimated through omega, for each of the Big Five factors before and after *AV*-adjustment. We found support for this hypothesis such that there was a higher internal consistency for the *AV*-adjusted scores indicating better measurement properties.

Hypothesis 2 was also confirmed: the discrimination parameters based on the AV-adjusted scores were larger, while the thresholds spanned a wider range. In sum, the *AV*-adjustment increased the overall test information, adding power and precision to the test. This finding again suggests that the *AVs* are very beneficial from a psychometric perspective.

To test Hypothesis 3, we assumed that the *AV*-adjusted scores provided clearer support for the Big Five Factor structure of personality, compared to the original scores, which were DIF-affected items and failed to show measurement invariance. Overall, we found the *AV*-adjusted scores better predict the estimated level of the latent factor, including more reliable and factorially-pure scales aligned with the Big Five Factor structure. They are therefore more in line with previous findings (mostly based on exploratory factor analysis) regarding the Big Five factor structure (notably obtained with samples that are more homogenous culturally than the two chosen for the present investigation). It has to be noted that Model #4, based on the *AV*-adjusted scores for the Filipino sample, did not have acceptable fit. However, with personality data, a confirmatory factor analysis is not always desirable due to specific characteristics of the data (e.g., model complexity; Hopwood and Donnellan, 2010; Fischer, 2014). In general, the confirmation of the third hypothesis is also in line with the findings of the graded response model (Hypothesis 2). In conclusion, all psychometric characteristics are improved following the *AV*-adjustment: *AV*-adjusted items that are DIF-free appear to improve comparability across countries.

For the final hypothesis (Hypothesis 4), we evaluated test-criterion relationships by examining correlations between the original and *AV*-adjusted scores with two external outcome measures. In our results, Conscientiousness, Agreeableness, and Openness, based on both the original scores and the *AV*-adjusted scores, correlated negatively with counterproductive work behavior, as expected. Likewise, Neuroticism had a slightly positive correlation and Extraversion was unrelated before the *AV*-adjustment. However, the magnitude of these correlations was significantly lower when using the *AV*-adjusted scores. Both studies showed weak non-significant correlations of the Big Five with satisfaction with life. Looking at these correlations, we see that relations of satisfaction with life with Openness and Conscientiousness are significantly different depending on whether *AV*-adjustment is used or not. Agreeableness, Neuroticism, and Extraversion showed no significant variation before and after the *AV*-adjustment. Especially unexpected is the negative correlation of satisfaction with life with Conscientiousness after the *AV*-adjustment. However, these results are in line with previous findings in Iranian samples (Hosseinkhanzadeh and Taher, 2013). Notably, the unexpected drop in correlation magnitude of the Big Five with both outcome variables is consistent with the findings of Ham and Roberts (2015) who found a similar reduction in correlation with outcome values after applying the *AV* methodology.

One possible explanation for a fairly systematic reduction in these correlations is as follows. *AVs* were only applied on the personality items and not on the outcome measures *per se*; that is, DIF was only corrected for in personality. Thus, this method triggered a decrease in the covariance as the comparison is made between DIF-free items and items that are still DIF-affected. A further explanation is that the individual's ranking of personality substantially moved after the *AV*-adjustment, since the correlation between the original self-report dimensions and the *AV*-adjusted dimensions were around *r* = 0.38 to 0.64. Moreover, the stability of the fairly small correlations is somewhat questionable. Some correlations drop to a non-significant level if countries are examined separately. Thus, adjusting both the predictor and outcome variables is worth considering.

### Limitations

*AVs* are a strong theoretical and practical tool for accommodating DIF. However, this tool faces some general limitations worth mentioning. First, Buckley (2008) demonstrates that context effects can bias the vignette response, as do the order of the vignettes relative to the self-report. We did not test for order effects in the current study (*AV*s came before the BFI-44) largely because of concerns by the local administration of having multiple forms. Nevertheless, this concern is worthy of consideration.

Jürges and Winter (2013) showed the importance of vignette equivalence by highlighting that vignette ratings are somewhat sensitive to the sex and age of the hypothetical person described in the vignette. Likewise, participants may apply different thresholds for male and female hypothetical scenarios affecting response consistency (Kapteyn et al., 2007). Randomization can be used as a technique to neutralize violations of response consistency (Chan et al., 2015). In our study, we randomized the sex of the possible descriptions, as well as the names, which could also show some relation to age groups. However, we were unable to randomize the order of the AVs, which would have allowed us to address contextual effects.

Our study was limited in the number of countries assessed, the regions surveyed, and the sample size within each country. In particular, this limitation prevented us from using the parametric approach (King et al., 2004) to analyze the *AVs*; hence, we applied the non-parametric approach for both samples. The non-parametric approach shows limitations when dealing with order violations: inconsistencies are grouped and the non-parametric solution can only deal with scalar values, resulting in a loss of information (Paccagnella, 2013). Hence, all non-systematic order violations in our study were treated as ties, leading to a loss of information. Most studies experience some degree of order violation. He et al. (2017) reported order violations ranging from 3 to 13% for facets of Conscientiousness, with the exception of the facet industriousness, which had an order violation of 30%. They concluded that all *AV*-sets worked well, even though presenting vignettes with two levels, instead of vignettes with three levels, expect the *AV*-set for the Conscientiousness facet of industriousness, which was hence excluded from the analysis (He et al., 2017). In our study, at least two Big Five factors showed more than 10% order violations. It has been argued that it is not problematic for later interpretation when vignettes are ordered incorrectly because a participant experienced the *AV*s differently based on their circumstances (Wand, 2013). Any kind of disagreement on the actual vignette order should be explored as a possible design problem and as an indication of a poor vignette. Hence, the quality of our Openness vignettes, where order violations ranged from 10% (Philippines) to 23% (Rwanda), can be improved and a revision of this vignette set, as well as the neuroticism vignettes (15% order violation in Rwanda and 18% on the Philippines), should be pursued in future studies.

The representativeness of the results for both countries may be restricted to the specific regions of the countries where the data was collected and may be slightly more representative of females, as both samples include a high percentage of women.

### Future considerations

Based on these considerations, future studies should include larger samples, more countries, and more regions within countries, as this will allow the researcher to use the parametric approach. Those results could then be compared to results using a non-parametric approach. Using *AV*-adjustment in countries where the Big Five Factor structure is replicated and well-established would allow for an interpretation of mean-shifts in trait scores after the *AV*-adjustment. Also, it is important to note that the Big Five Factor structure was replicated quite well in Rwanda after the *AV*-adjustment. However, this result should be replicated in future research. Future studies should also further explore test-criterion relationships after *AV*-adjustment, particularly with a wider range of criterion variables, given our work in this area was limited.

Our findings are especially relevant for researchers interested in alternative or competing methods to measure personality. Our study provides insights concerning the robustness and the universality of the Big Five Personality factors. Influential articles describe the Big Five as a psychometrically sound measure that can be applied in different countries and cultures (e.g., McCrae and Terracciano, 2005; Schmitt et al., 2007). Self-report measures of the Big Five, which have their origin in a lexical approach, are based on principal component analysis with a varimax rotation, meaning the five factors are kept orthogonal (e.g., Tupes and Christal, 1961). However, if the fit of the model is assessed with confirmatory factor analysis or item response theory, this structure often has a poor fit to the data and insufficient psychometric properties (Olaru et al., 2015). The confirmatory models based on the original self-report scores in our study supports these concerns. The *AV*-adjustments leading to DIF-free scores show a promising solution toward a psychometrically sound measurement with interpretable, reliable, and factorially-pure scales.

AV-adjustment is especially relevant today given personality research is facing a debate on the comparability of results based Likert-scale response options, ranging from issues with cross-cultural comparison (He et al., 2017) to comparability between genders (Weisberg et al., 2011). The application of an external benchmark, like the *AVs*, for all Big Five dimensions is not only of interest for correcting cross-cultural bias, but rather for any kind of bias between different groups (e.g., men and women).

The *AV*s provided in Table 2 can be applied not only in a research context (e.g., global workforce, developmental and educational research and policymakers), but also in occupational context. For example, large international companies that base their application and selection process on assessed cognitive and non-cognitive skills can apply vignettes in order to minimize cross-cultural bias in the assessment of non-cognitive skills.

## Conclusion

This study is one of the first to use *AV* methodology to adjust all Big Five dimensions in more than one country (cf. Primi et al., 2016). In order to use *AVs*, we tested and showed that we met the basic measurement assumptions. The literature regarding the utility of *AV*-adjustment for the assessment of personality is mixed, with researchers finding either the method is not necessary or very beneficial. We showed that personality self-reports in Rwanda and the Philippines are affected by DIF and improved with an *AV*-adjustment. Even though the trait-level means for the original and *AV*-adjusted scores were not drastically different, several psychometric characteristics were improved when *AV*-adjusted scores were used. In the end, the DIF-free scores led to more reliable, powerful, and precise scales that are in line with the Big Five Factor structure. However, test-criterion relations were somewhat reduced after the *AV*-adjustment—a finding discussed above. This finding notwithstanding, we argue that the *AV*-adjustment makes personality across countries more comparable and offers a possible solution to cross-cultural comparison problems.

In sum, this study and its results act as an important step toward explaining and handling cross-cultural comparability problems. Overall, *AVs* provide a useful external benchmark for adjusting self-report scores when measuring personality. Future studies should consider implementing similar adjustments to the assessment of other psychological constructs.

## Author contributions

SW contributed to the conception of the study, the data analysis, and the interpretation of data for the work. SW drafted the article and finalized the version for publication. RR contributed to the conception and design of the study, and the acquisition and interpretation of the data. RR also edited the final manuscript. SW and RR agreed to be held accountable for all aspects of the work and ensure that questions related to the accuracy or integrity of any part of the work will be appropriately investigated and resolved.

### Conflict of interest statement

The authors declare that the research was conducted in the absence of any commercial or financial relationships that could be construed as a potential conflict of interest.
