# Supplementary material for: Using Anchoring Vignettes to Adjust Self-Reported Personality: A Comparison Between Countries
Source: Front Psychol. 2018 Mar 14;9:325. doi: 10.3389/fpsyg.2018.00325 (PMC5861527; doi:10.3389/fpsyg.2018.00325)
Supplement: Supplementary file 1 [file Table1.docx]

Supplementary Material

**Using Anchoring Vignettes to Adjust Self-Reported Personality across Countries**

Selina Weiss^*^, Richard D. Roberts

*** Correspondence:** Selina Weiss: [Selina.weiss@uni-ulm.de](mailto:Selina.weiss@uni-ulm.de)

# Supplementary Tables

**Table S1.** R-Scripts examples for Conscientiousness items and the corresponding anchoring

-vignette set

|  | R-Syntax |
| --- | --- |
| Calculating lower and upper bound of DIF-free items (based on the non-parametric approach) | con2 <- anchors(BFI_8r ~ AV_1+AV_2+AV_3, data, method="C")  summary(con2)  datacon2 <- insert(data, con2)  write.table(datacon2, file="datacon2.txt",sep="\t") |
| Analyzing the order of the anchoring vignette sets (correct order, ties and inconsistencies ) | order_con <- anchors.order(~AV_1+AV_2+AV_3, data)  summary(order_con, top=10,digits=3)  barplot(order_con) |
| Entropy | sub8 <- list(self = BFI_8r ~ 1, vign = cbind (AV_1,AV_2,AV_3) ~ 1,  cplor=~Country)  ent8 <- anchors(sub8, data = data, method="C", combn=TRUE)  summary (ent8, digits=3)  plot(ent8) |
